# Supplementary material for: Increased Apoptotic Activity in Low-Risk Myelodysplastic Syndrome
Source: J Clin Med. 2022 Aug 7;11(15):4604. doi: 10.3390/jcm11154604 (PMC9369950; doi:10.3390/jcm11154604)
Supplement: Supplementary file 1 [file jcm-11-04604-s001.zip › jcm-1831357-supplementary.pdf]

Journal of
Clinical Medicine

MDPI

Table S1. Patient characteristics in the patients with ICUS (*n* = 37) [5].

Variables	Number of Patients (%)
Age at diagnosis,	
median (range)	66 (31–83)
Sex	
Male	15 (40.5)
Female	22 (59.5)
Neutropenia (ANC < 1800/mm^3)	28 (70.0)
Anemia (Hemoglobin < 10 g/dL)	30 (75.0)
Platelets (<100,000/ mm^3)	21 (52.5)
Lineage involved of cytopenia	
Uni-lineage	8 (21.6)
Bi-lineage	22 (59.5)
Tri-lineage	7 (18.9)
Grade of cytopenia	
1	5 (13.5)
2	8 (21.6)
3	21 (56.8)
4	3 (8.1)

Abbreviations: ICUS, idiopathic cytopenia of undetermined significance; ANC, absolute neutrophil count.

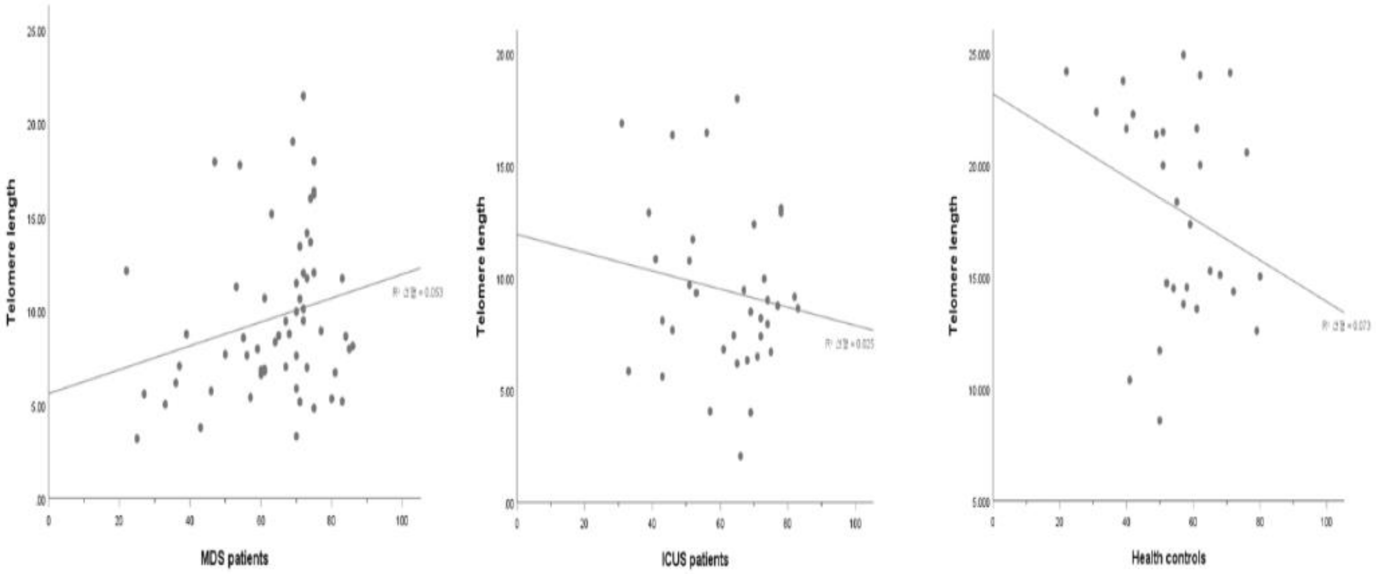

Figure S1. Telomere length according to age.

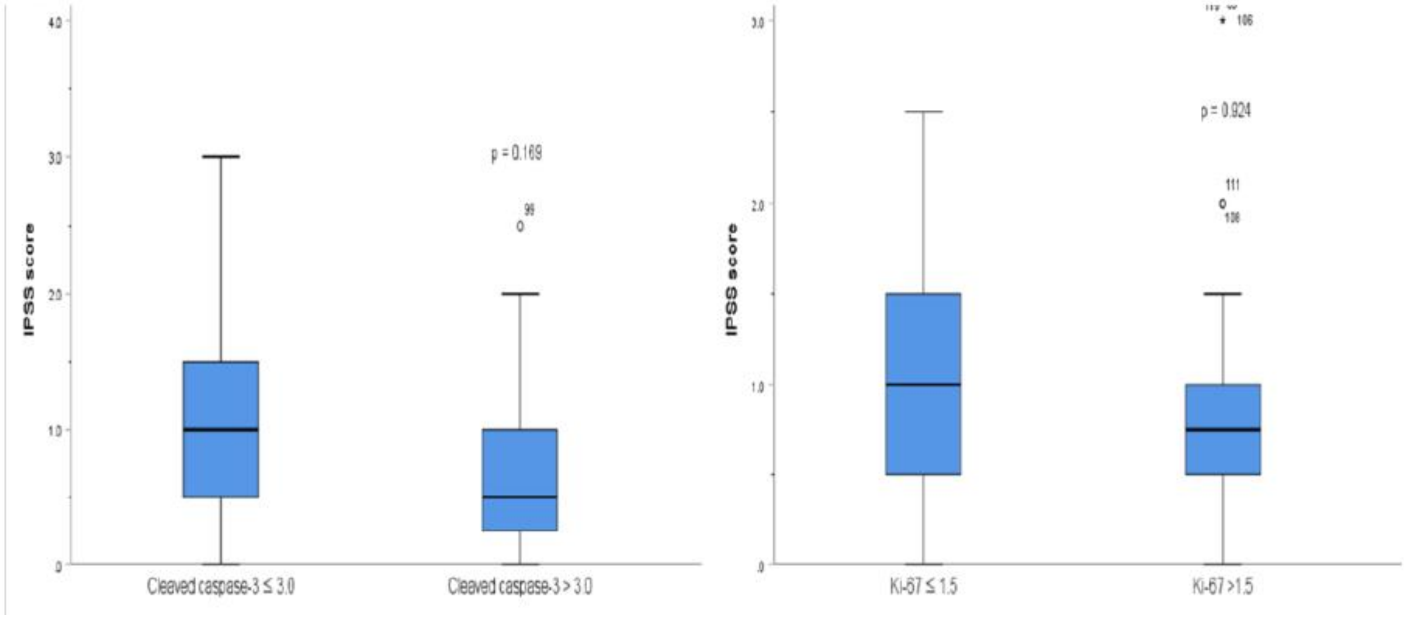

Figure S2. IPSS score according to cleaved caspase-3 and Ki-67. IPSS, International scoring prognostic system.
